# Supplementary material for: Content-rich biological network constructed by mining PubMed abstracts
Source: BMC Bioinformatics. 2004 Oct 8;5:147. doi: 10.1186/1471-2105-5-147 (PMC528731; doi:10.1186/1471-2105-5-147)
Supplement: Additional File 5 — The original Chilibot query results of the term "long-term potentiation (LTP)" and 22 other terms, limiting the latest references analyzed to the years 1990, 1995, 2000, and 2004. [file 1471-2105-5-147-S5.bz2 › chilibotAdditionalFile5/ltp2000/html/PLC_PKC.html]

 


 **PLC** and **PKC** 
  
Found 470 abstracts in PubMed,  **30 abstracts were retrieved and analyzed**.  


---

 Search Google  |
 PDF files only 
|  EDU domain only 

---

**Interactive relationship** (e.g. stimulation, inhibition, etc)

- Results presented here also show that exogenous addition of Bacillus cereus PC  **PLC**  activates PC hydrolysis,  **PKC**  and PLD in all three murine cell lines.  Ref: 10936719 Comp Biochem Physiol BComp Biochem Physiol B, 2000
- Results presented here also show that exogenous addition of Bacillus cereus PC  **PLC**  activates PC hydrolysis,  **PKC**  and PLD in all three murine cell lines.  Ref: 11007187 Comp Biochem Physiol B Biochem Mol Biol, 2000
- Short term activation of  **PKC**  had no effect on receptor binding or effector enzyme adenylyl cyclase or  **PLC**  beta activity.  Ref: 11003572 Am J Physiol Cell Physiol, 2000
- In turn, activation of this atypical  **PKC**  leads to the stimulation of phosphatidylcholine phospholipase C PC  **PLC**  and acidic sphingomyelinase ASMase.  Ref: 11001916 Blood, 2000
- These findings indicate that the distinct temporal profiles of  **PLC**  activation by P2Y 2 receptors and mAChR may arise from the differential activation of  **PLC**  beta1 and  **PLC**  beta3 by the receptors, coupled with a selective  **PKC**  mediated negative feedback mechanism that targets  **PLC**  beta3 but not  **PLC**  beta1.  Ref: 10995776 J Biol Chem, 2000
- Our results suggest that  **PLC**  are responsible, via a calcium and protein kinase C  **PKC**  dependent pathway, for p60Src activation and tyrosine phosphorylation of the p60Src substrate, cortactin.  Ref: 10975856 J Immunol, 2000
- Finally, Purkinje like cells  **PLC**  in the VCN, which express only  **PKC**  delta in control rats, began to express  **PKC**  gamma after deafferentation, correlated with increased expression of calbindin D28k in  **PLC** .  Ref: 10962178 Hear Res, 2000
- These data suggest that both D2R agonists and ethanol activate  **PLC**  via a trimeric G protein leading to production of diacylglycerol with subsequent activation and translocation of delta and epsilon  **PKC** .  Ref: 11125036 Mol Pharmacol, 2001
- EGF treatment did not modify the phosphorylation of the isoform of phospholipase C  **PLC** , and inositol trisphosphate IP3 and intracellular calcium levels, but it induced an increase in  **PKC**  activity.  Ref: 10918075 Exp Physiol, 2000
- The  **PKC**  mediated phosphorylation of  **PLC**  beta3 diminishes the interaction of Galpha q 11 with  **PLC**  beta3, thereby contributing to the termination  **PLC**  beta3 activity.  Ref: 10995776 J Biol Chem, 2000
- The  **PKC**  specific inhibitors bisindolylmaleimide and Gö 6976 abolish insulin induced PLD2 activation in HEK 293 cells co expressing the insulin receptor,  **PLC**  gamma and PLD2, confirming that not only PLD1, but PLD2 as well, is regulated in a  **PKC**  dependent manner.  Ref: 11042115 Biochem J, 2000
- Moreover, deficiency in fyn dependent  **PLC**  gamma1 catalytic activity may contribute to reduced  **PKC**  alpha dependent ERK activation.  Ref: 11093170 Eur J Immunol, 2000
- D2R agonist and ethanol induced translocation of delta and epsilon  **PKC**  share a common pathway that is blocked by pertussis toxin and requires phospholipase C  **PLC**  activity.  Ref: 11125036 Mol Pharmacol, 2001

**Parallel relationship** (e.g. studied together, co-existance, homology, etc.)

- We have previously reported that Fas cross linking resulted in the activation of phosphatidylcholine specific phospholipase C PC  **PLC**  and the subsequent activation of protein kinase C  **PKC**  and phospholipase D PLD in A20 cells.  Ref: 11007187 Comp Biochem Physiol B Biochem Mol Biol, 2000
- the obtained data suggest that an activation of alpha1A adrenoceptors, presence in WAT, by agonist and or neurotransmitter may increase the glucose uptake via  **PLC**   **PKC**  pathway and the activation of PI 3 kinase.  Ref: 11111846 Auton Neurosci, 2000
- Taken together, short term activation of GPCRs can induce a strong and long lasting sensitization of  **PLC**  stimulation by RTKs, a process apparently involving G i derived Gbetagammas as well as increases in intracellular calcium and activation of a  **PKC**  isoenzyme, most likely  **PKC**  alpha.  Ref: 10908568 J Biol Chem, 2000
- We demonstrate that  **PKC**  rather than protein kinase A mediates the G protein coupled receptor induced phosphorylation of  **PLC**  beta3.  Ref: 10995776 J Biol Chem, 2000
- On the other hand, both H 2 O 2 30 microM and ATP 100 microM induced phasic contractions were also attenuated, to different degree, by inhibitors of several enzymes including  **PLC** ,  **PKC** , PLA 2 and cyclooxygenase.  Ref: 10963730 Cardiovasc Res, 2000
- TNF alpha and IL 1beta mediated responses were modulated by  **PLC** , calcium,  **PKC** , and tyrosine kinase associated with cell proliferation in TSMCs.  Ref: 10864897 Br J Pharmacol, 2000
- Several CD2 pathway components regulated by p59 fyn have been identified including phospholipase C gamma1  **PLC**  gamma1, Vav, protein kinase C theta isoform  **PKC**  theta, docking protein Dok, focal adhesion kinase FAK and Pyk2.  Ref: 11093170 Eur J Immunol, 2000
- When the effect of anti Fas monoclonal antibody on  **PKC**  and PC  **PLC**  activities in Fas expressing clones were investigated, the activation of  **PKC**  and PC  **PLC**  was detected only in A20 clones.  Ref: 11007187 Comp Biochem Physiol B Biochem Mol Biol, 2000
- UTP induced increased PGE 2 release was demonstrated by a concomitant increase in COX 2 protein expression, and was decreased by inhibitors specific for phosphatidylinositide phospholipase C PI  **PLC** , CaMK, protein kinase C  **PKC** , nuclear factor kappa B NF kappaB or COX 2.  Ref: 10864883 Br J Pharmacol, 2000
- the inhibitory effect of imipramine depends partly on the  **PLC**   **PKC**  pathway.  Ref: 10903980 Br J Pharmacol, 2000
- Furthermore, inhibition of conventional protein kinase C  **PKC**  isoenzymes and chelation of intracellular calcium suppressed the sensitization process, while overexpression of  **PKC**  alpha... further enhanced the M 2 mAChR induced sensitization of  **PLC**  stimulation.  Ref: 10908568 J Biol Chem, 2000
- These data suggest that, in rat cerebrocortical synaptosomes, the 1S, 3R ACPD sensitive mGluR is coupled to PLD through a mechanism that is independent of both  **PKC**  and PI  **PLC** .  Ref: 11053224 Br J Pharmacol, 2000
- In vitro phosphorylation of RGS2 by  **PKC**  decreased its capacity to attenuate both GTP and GTPgammaS stimulated  **PLC**  betat activation, with the extent of attenuation correlating with the level of RGS2 phosphorylation.  Ref: 11063746 J Biol Chem, 2001
- Our data show that EGF regulates amino acid uptake, via  **PKC**  and without  **PLC**  activation, only in the last period of chick embryo hepatocyte development.  Ref: 10918075 Exp Physiol, 2000
- When the effect of anti Fas monoclonal antibody on  **PKC**  and PC  **PLC**  activities in Fas expressing clones were investigated, the activation of  **PKC**  and PC  **PLC**  was detected only in A20 clones.  Ref: 10936719 Comp Biochem Physiol BComp Biochem Physiol B, 2000
- We have previously reported that Fas cross linking resulted in the activation of phosphatidylcholine specific phospholipase C PC  **PLC**  and the subsequent activation of protein kinase C  **PKC**  and phospholipase D PLD in A20 cells.  Ref: 10936719 Comp Biochem Physiol BComp Biochem Physiol B, 2000
- It was concluded that GYIRFamide stimulates mechanical activity in F. hepatica through a G protein, via a  **PLC**  and  **PKC**  dependent second messenger pathway.  Ref: 11080073 Am J Physiol Regul Integr Comp Physiol, 2000
- To examine the pathway of  **PKC**  activation, chondrocyte cultures were treated with E 2 in the presence or absence of genistein an inhibitor of tyrosine kinases, U73122 or D609 inhibitors of phospholipase C  **PLC** , quinacrine an inhibitor of phospholipase A 2 PLA 2, and melittin an activator of PLA 2.  Ref: 11070350 J Steroid Biochem Mol BiolJ Steroid Biochem Mol Biol, 2000
- **PLC**  and PI3K, in addition to  **PKC**  and TK, are involved in the PAR 1 mediated dual responses.  Ref: 11065174 Life Sci, 2000
- This acceleration was found to be dependent on phospholipase C  **PLC**  and protein kinase C  **PKC**  activities and intracellular calcium.  Ref: 10974320 Neuropharmacology, 2000
- Inhibitors of phospholipase C  **PLC** , protein kinase C  **PKC** , calcium calmodulin dependent protein kinase CaMK and antagonists of the L type calcium channel also inhibit the corticosterone induced gene transcription.  Ref: 10903980 Br J Pharmacol, 2000
- OBJECTIVE AND DESIGN This study was aimed to evaluate the effects of two protein kinase C  **PKC**  inhibitors staurosporine and chelerythrine and one phospholipase C  **PLC**  inhibitor U73122 on basophil histamine release induced by anti IgE, N formyl methionyl leucyl phenylalanine FMLP, IL 3 and GM CSF.  Ref: 11071123 Inflamm Res, 2000
- We investigated the involvement of protein kinase C  **PKC**  in the termination of  **PLC**  beta activation induced by endogenous P2Y 2 purinergic receptors and transfected M 3 muscarinic acetylcholine receptors mAChR in Chinese hamster ovary cells.  Ref: 10995776 J Biol Chem, 2000
- In summary, we demonstrate that insulin treatment results in activation of both PLD1 and PLD2 in appropriate cell types when the appropriate upstream intermediate signalling components, i.e.  **PKC**  alpha and  **PLC**  gamma, are expressed at sufficient levels.  Ref: 11042115 Biochem J, 2000
- After incubation with  **PKC**  and  **PLC**  inhibitors, the stimuli were added and histamine release was measured by an automated fluorometric method.  Ref: 11071123 Inflamm Res, 2000
